# Single-use flexible ureteroscopes: practice patterns, attitudes, and preferences for next-generation concepts

**DOI:** 10.3389/fsurg.2024.1419682

**Published:** 2024-07-04

**Authors:** Bassel Salka, Jamsheed Bahaee, John Michael DiBianco, Jeff Plott, Khurshid R. Ghani

**Affiliations:** ^1^ University of Michigan Medical School, University of Michigan, Ann Arbor, MI, United States; ^2^ General Urology and Pelvic Health Center, Cleveland Clinic Akron, Akron, OH, United States; ^3^ Department of Urology, University of Florida, Gainesville, FL, United States; ^4^ Coulter Program, Department of Biomedical Engineering, University of Michigan, Ann Arbor, MI, United States; ^5^ Department of Urology, University of Michigan, Ann Arbor, MI, United States

**Keywords:** single use, ureteroscopy, urolithiasis, technology, survey

## Abstract

**Background:**

Single use flexible ureteroscopes (su-fURS) have emerged as an alternative to reusable flexible ureteroscopes (r-fURS) for the management of upper urinary tract calculi. However, little is known about urologist usage and attitudes about this technology. Through a worldwide survey of endourologists, we assessed practice patterns and preferences for su-fURS.

**Methods:**

An online questionnaire was sent to Endourology Society members in January 2021. The survey explored current su-fURS practice patterns, reasons for/against adoption, and preferences for next generation models including developments in imaging, intra-renal pressure, heat generation, and suction. Responses were collected through QualtricsXM over a 1-month period from surgeons in North America, Latin America, Europe, Asia, Africa, and Oceania. The study was conducted according to the Checklist for Reporting Results of Internet E-Surveys (CHERRIES).

**Results:**

208 (13.9%) members responded to the survey. Most respondents (53.8%) performed >100 ureteroscopies per year. 77.9% of all respondents used su-fURS for less than half of all procedures while only 2.4% used su-fURS for every procedure. 26.0% had never used a su-fURS. Overall, usage was not influenced by a surgeon's geographic region, practice environment, or years of experience. Top reasons for not adopting su-fURS were cost (59.1%) and environmental impact (12.5%). The most desired improvements in design were smaller outer shaft size (19.4%), improved optics and vision (15.9%), and wireless connectivity (13.6%). For next generation concepts, the functions most commonly described as essential or important by respondents was the ability to suction fragments (94.3%) while the function most commonly noted as not important or unnecessary was incorporation of a temperature sensor (40.4%).

**Conclusions:**

su-fURS are not commonly used, even among urologists who perform a high number of fURS. The primary concern for adoption is cost and environmental impact. Suction capability was considered the most important future development.

## Introduction

Flexible ureteroscopy (fURS) is now commonly used for upper urinary tract calculi while URS has become the number one treatment modality for this in North America (1, 2). Single use flexible ureteroscopy (su-fURS) has emerged as an alternative to traditional reusable flexible ureteroscopy (r-fURS). Since the introduction of the LithoVue single use ureteroscope in 2016 (3), su-fURS has been marketed as a cost-effective alternative that eliminates many of the challenges presented by r-fURS. Reusable scopes are expensive devices that require inter-procedural disinfection (4), have durability issues after multiple uses (5), and require repair at variable time points (6–8). Designed to replicate the feel and operative functionality of r-fURS with the exception that surgeons throw the device away after surgery rather than set aside for disinfection, adoption of su-fURS can theoretically bypass these challenges while maintaining similar patient outcomes (9, 10).

However, little is known about the extent to which urologists have adopted this technology. With no international guidelines yet providing a recommendation on the topic, some urologists have expressed concerns about decreased image quality, increased costs, and environmental issues related to disposal as reasons to not embrace su-fURS as a new standard (11). Although su-fURS No study has yet evaluated urologist practice patterns and attitudes towards su-fURS at an international level. Learning more about the factors limiting urologist adoption of su-fURS and the functionality they would like to see in future concepts can help guide designs and innovations.

In this context, we distributed a survey to Endourology Society members to evaluate su-fURS practice patterns and attitudes. Specifically, the purpose of our study was to (1) Determine current usage of su-fURS, (2) Identify limiting factors to su-fURS adoption, and (3) Explore surgeon preferences for the development of future su-fURS concepts. We hypothesized that more industrialized nations (United States, Canada, etc.) would have greater adoption of su-fURS.

## Methods

An online survey investigating practices and attitudes towards su-fURS was sent to all members of the Endourology Society (approximately 1,500 members) in January 2021, through an email invitation. The survey was anonymous and answers confidential. The survey contained questions that focused on su-fURS irrigation practices including (1) participant demographics, (2) current practice patterns of su-fURS, (3) reasons for not adopting su-fURS, and (4) preferences for next generation models. The survey utilized a variety of question formats including multiple choice, optional free response, and ranking choice answers (Supplementary Material). The survey was conducted using the web-based QualtricsXM (Provo, UT). An introductory email containing a brief introduction and hyperlink invited Endourology Society members to participate in the survey with a second reminder email sent two weeks later. The survey remained open for 5 weeks. A monetary gift award was offered as part of this study to one randomly selected respondent to improve participation. The survey received Institutional Review Board approval at the University of Michigan and was conducted according to the Checklist for Reporting Results of Internet E-Surveys (CHERRIES) to ensure the quality of survey design, administration, and data reporting.

## Results

### Demographics

A total of 208 (13.9%) members responded to the survey. Most respondents (65%) practiced in North America (United States and Canada) or Europe. 55.8% of surgeons practiced at a university hospital setting while 23.6% worked in a community/private practice. 64.2% of respondents had been practicing for greater than 11 years.

### Current practice patterns of su-fURS

**Figure 1:**
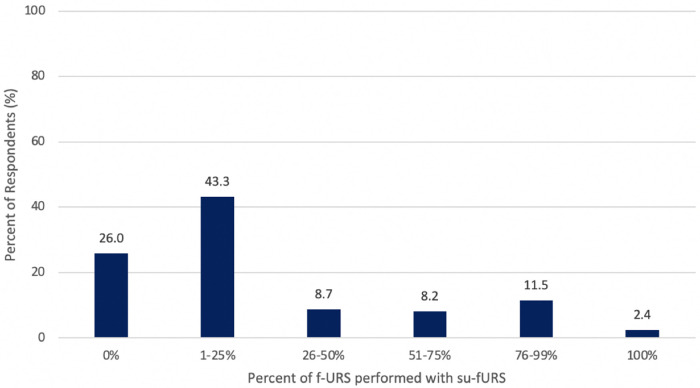
Percentage of flexible ureteroscopy (f-URS) that surgeons performed with a single-use ureteroscope (su-fURS).

Most surgeons (53.8%) performed more than 101 fURS annually, with 26.4% performing greater than 200. The majority (77.9%) used su-fURS for less than half of all procedures while only 2.4% used su-fURS for every procedure (Figure 1). 26.0% of all respondents responded as never using a su-fURS before. For those that used su-fURS, 67.5% never re-sterilized the device while 13.6% did so routinely. The most common situations for utilization of su-fURS were lower pole kidney stones (15.7%), stone size >2 cm (10.8%), and horseshoe kidney (10.1%). No significant differences existed in frequency of su-fURS use between surgeons in different geographic regions, practice environments, or with varying years of experience (*p* > 0.05).

### Reasons for not adopting su-fURS

The most common limiting factors to su-fURS adoption were cost (59.1%) and environmental impact (12.5%). In a follow-up free text option to expand on reasons for not adopting the technology, 3 respondents explained that challenges with insurance coverage contributed to cost concerns. For respondents who used su-fURS, 59.1% reported no change in operating room (OR) time, 34.4% reported a decrease in OR time, and 6.5% reported an increase in OR time.

### Preferences for next generation models

**Figure 2:**
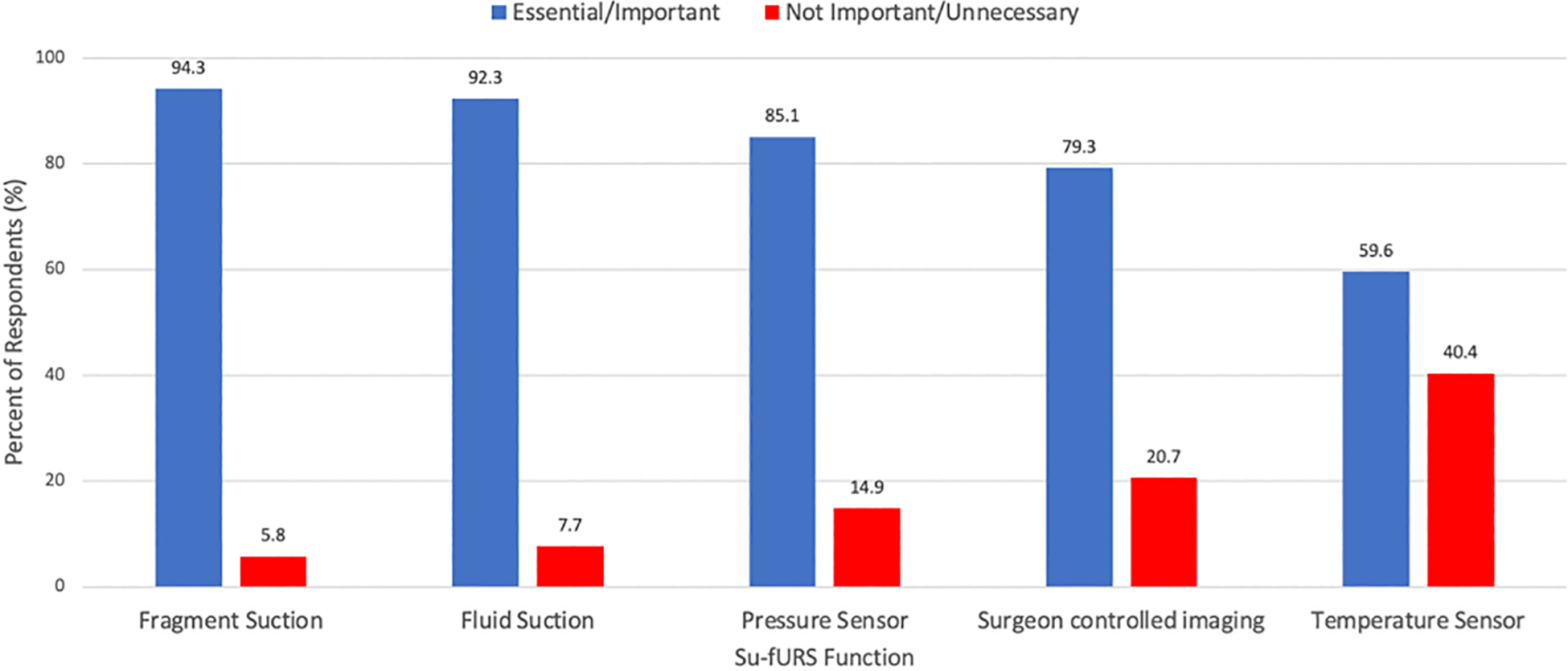
Surgeons’ responses to appraisal of future concepts in single-use ureteroscopy (su-fURS) functional ability: is it essential/important or not important/unnecessary?

The features of su-fURS identified as most important was image quality (38.5%), deflection ability in the lower pole (22.1%), and size of scope (19.2%). The functions most described as “Essential” or “Important” by respondents was the ability to suction fragments (94.3%) and fluid (92.3%) while most commonly described as “Unnecessary” or “Not important” was incorporation of a temperature sensor (40.4%) and surgeon-controlled imaging (20.7%) (Figure 2). Most respondents (78.9%) preferred the working channel location on the dorsal surface vs. the posterior surface, near the deflection handle. The most desired improvements in su-fURS design were smaller outer shaft size (19.4%), improved optics and vision (15.9%), and wireless connectivity to image monitor (13.6%).

## Discussion

We conducted an online survey study to understand endourologist practices and attitudes toward su-fURS technology. Although most of our respondents performed a high volume of ureteroscopies, very few have adopted su-fURS. A quarter of surgeons reported as never having used a su-fURS before. This usage was not influenced by a surgeon's geographic region, practice environment, or years of experience. The most cited reason for not adopting the technology was cost and environmental impact. When queried on the most desired improvements in su-fURS design, smaller shaft size and improved optics were the most common responses. The most valued function in s-fURS was the ability to suction fragments and fluid. Our hypothesis that urologists in more industrialized nations would have greater adoption of su-fURS was refuted by no statistical differences in su-fURS adoption regardless of geographic location, ureteroscopy experience, and/or practice setting.

Our study is not the first to explore su-fURS utilization amongst urologists. A survey completed by 114 members of the European Association of Urology young academic urologists and uro-technology groups in 2022 found that only 2.7% of respondents used su-fURS more than 80% of the time, with 59.4% not using su-fURS at all (12). In addition to showing progression of su-fURS utilization in this more contemporary survey, we are the first to explore reservations urologists may have to adopting the technology. Identification of cost and environmental impact as the two primary concerns urologists sets the precedent for discussion on whether these factors are substantiated and provides context to our findings of relatively low adoption of su-fURS.

A number of studies have evaluated the overall cost of su-fURS compared to r-fURS (4, 13, 14). A recent systematic review identified studies comparing the price of su-fURS and r-fURS and found that in the few studies that have reported costs, a local and international variation exists in acquisition of the scopes as well as maintenance and repair costs for r-fURS (15). However, they concluded that a partial overlap exists in cost between su-fURS and r-fURS after all are accounted for, but that it is important to know the precise caseload, repair bills, and added expenses when deciding on acquisition. The primary factors contributing to r-fURS cost variation is cost of repair, which is generally compared to the higher acquisition costs associated with su-fURS. A further meta-analysis quantified the economic burden of repair costs and found a repair rate of 6.5% in r-fURS, equivalent to about 15 cases between repairs and 441 USD per procedure (16). Reported factors influencing the occurrence of breakage include the number of surgeons who have access to the scope, endourological vs. non-endourological centers, university vs. private hospital, and the degree of training of the personnel involved in the use or reprocessing of the scopes (17).

There is limited literature examining the environmental impact of s-fURS compared to r-fURS. An analysis of the typical life cycle of su-fURS found the total carbon footprint per case of one of the most popular su-fURS was 4.43 kg of CO_2_, compared to 4.47 kg of CO2 for r-fURS, concluding that the environmental impact of r-fURS and su-fURS are comparable (18). However, these results are based on a number of assumptions including the number of times the scope is used, the number of times between repair, the type of su-fURS used, and more. More research on the environmental impact of URS is required (19).

Given the value our respondents place on image quality and mobility of URS, the finding that few urologists in our survey are concerned about the functional quality and related outcomes of su-fURS is noteworthy. Many studies have evaluated the performance of su-fURS and report similar imaging quality (20–22), usability (23–25), and outcomes (9, 26) compared to r-fURS. One meta-analysis comparing su-fURS and r-fURS found no significant differences in procedure duration, stone size, stone clearance and complication rates (9). It concluded that su-fURS demonstrates comparable efficacy with r-fURS in treating renal calculi. These findings are shared by the results of our survey that found no significant changes in procedure duration with su-fURS and a satisfaction with the image quality, deflection ability in the lower pole, and size of scope.

Our findings suggest that most urologists are not apprehensive about the functionality of su-fURS, but rather about the cost and environmental impact of the devices. The evidence suggests that in some situations, su-fURS may be a better alternative to r-fURS in these domain (15, 18). As such, efforts for su-fURS adoption should focus on addressing concerns about cost and environmental impact. Urologist perspectives are key to adoption and should also be considered for next generation concepts of ureteroscopy. The next generation concepts identified in our study such as adding the ability to suction fragments/fluid, placing the working channel at the dorsal surface, decreasing the size of the outer diameter, and improved optics and vision should all be considered to further develop su-fURS. Indeed efforts are underway by multiple companies in this regard.

Our study is the largest to evaluate su-fURS practice patterns, perspectives, and preferences for future concepts. However, certain limitations are acknowledged. First, our response rate of 13.9% represents a small proportion of urologists. Although this is consistent with survey studies in Endourology (27–31), the response rate may introduce the risk of potential biases posed by urologists' geographic location, ureteroscopy experience, and/or practice setting. In addition to our survey being sent evenly to all Endourology Society members independent of practice setting and urologist characteristics, our results showed no difference in adoption of sURS between these subgroups. Secondly, the respondents were members of the Endourology Society, and therefore a highly specialized cohort that may not be generalizable to general urologists. However, this group of endourology leaders will likely influence the trajectory of ureteroscopic innovation, and therefore be representative of future generations of non-specialized urologists. Lastly, it is possible that we may have introduced a questionnaire bias by failing to ask other important questions or by not providing sufficient answer choices. We worked to minimize this bias by designing the survey using a research team involved in endoscopic technology engineering and immersed in developments in the field (32). There is also concern for reporting bias, in which respondents say one thing, and do another thing in clinical practice.

Limitations notwithstanding, our survey is important because it provides insight to the use and apprehensions urologist have for su-fURS. By querying a group of urologists across 6 continents, levels of experience, and practice settings, this study provides a generalized view of su-fURS reception internationally. We observe a relatively low adoption of this technology, and advocates for su-fURS now have a framework for what the concerns are. Additionally, a new evidence base is available for designers of next concept models in ureteroscopy that can improve surgeon effectiveness in the operating room. Future directions include research on the hurdles urologists may face in implementation of this new technology. The majority of our respondents hailed from North America and Europe, regions with highly developed infrastructure that may yield different cost considerations than developing countries (33). Other directions of research include exploring insurance considerations in su-fURS coverage and the unique challenges of an academic vs. private practice. For example, the 2023 introduction of the Traditional-Pass-Through payment category in the United States for Medicare patients in the outpatient hospital setting impacts reimbursement for the utilization of su-fURS and can influence future practice patterns (34). Efforts at future surveys should focus on increasing the number of respondents to best represent a diverse group of practice patterns.

In summary, while fURS procedures are common amongst endourologists, su-fURS is only regularly utilized by a small minority. The primary concerns endourologists have in adopting this new technology is increased cost and environmental impact. A more robust evidence base is required to substantiate these concerns. Urologist preferences for improvement on current models include reducing the shaft size, improving the optics, and adding wireless connectivity. Preferences for future concepts include fragment and irrigation suction capabilities.

## Data Availability

The raw data that support the conclusions of this article are available from the corresponding author upon reasonable request.
