# Supplementary material for: Single-use flexible ureteroscopes: practice patterns, attitudes, and preferences for next-generation concepts
Source: Front Surg. 2024 Jul 4;11:1419682. doi: 10.3389/fsurg.2024.1419682 (PMC11254690; doi:10.3389/fsurg.2024.1419682)
Supplement: Supplementary file 1 [file Table1.docx]

**APPENDIX - Survey**

**Attitudes and practices of single-use flexible ureteroscopes and next-generation technologies**

**Question 1: Where do you practice?**

- United States
- South America
- Europe
- Africa
- Oceania
- Asia
- Middle East
- Canada
- Mexico
- Central America

**Question 2: Primarily what environment do you practice in?**

- University hospital/setting
- Community/Private practice
- Combination university and private practice
- Government facility (VA, military base)

**Question 3: How many years have you been practicing?**

- <5
- 5-10
- 11-15
- 16-20
- >20

**Question 4: How many flexible ureteroscopy cases do you perform every year?**

- <25
- 25-50
- 51-100
- 101-200
- >200

**Question 5 (*Only appears to respondents that choose “<25” for question 4):* To the best of your knowledge, why does your department perform this number of flexible ureteroscopies every year? (Choose all that apply)**

- Lack of equipment
- Lack of patients
- Lack of experience with this technique
- Other[Describe]

**Question 6: Do you use flexible ureteroscopes with a dual-working channel?**

- Yes
- No

**Question 7 (*Only appears to respondents that choose “No” for question 6)*: Why do you not use a flexible ureteroscope with a dual-working channel? (Choose all that apply)**

- Never considered using it
- Do not see value in dual-channel scope
- Difficult to acquire equipment
- Increased outer shaft size
- Other[Describe]

**Question 8: In your experience, what is the most frequent type of damage to the reusable flexible ureteroscope? (Choose one)**

- Loss of deflection
- Optical damage (loss of vision)
- Working channel damage
- Laser damage to the scope
- Pressure leak test failure
- Other[Describe]

**Question 9: What proportion of your flexible ureteroscopies are performed with a single-use (disposable) flexible ureteroscope?**

- 0%
- 1-25%
- 26-50%
- 51-75%
- 76-99%
- 100%

**Question 10: Does your facility re-sterilize and re-use single-use (disposable) flexible ureteroscopes?**

- Routinely
- Sometimes
- Never

**Question 11: The following are factors that could limit your use of single-use (disposable) flexible ureteroscopes. Please rank the factors below from the most important limiting factor (rank number #1) to least important (rank number #7) (Drag the factors in the order you prefer)**

- Environmental impact
- Functionality in comparison to reusable flexible scopes
- Availability
- Cost
- Size of scope (outer shaft F)
- Image quality
- Weight

**Question 12: Are there any other factors limiting your use of single-use (disposable) flexible ureteroscopes beyond the list provided in the previous question?**

- No
- Yes[Describe]

**Question 13: Which of the following clinical scenarios would you consider using a single-use (disposable) flexible ureteroscope? (Choose all that apply)**

- Stone <1cm
- Stone 1-2cm
- Stone >2cm
- Lower pole kidney stone
- Patient with ileal conduit
- Patient with history of urinary tract infection
- Immunocompromised patient
- Caliceal diverticular stone
- Horseshoe kidney
- Working with resident/trainee
- Endoscopic combined intrarenal surgery
- Antegrade ureteroscopy
- All clinical cases
- Other[Describe]

**Question 14: Please rank the following characteristics of a single-use (disposable) flexible ureteroscope in order of importance to you. #1 being the most important factor, and #10 being the least important (Drag the factors in the order you prefer):**

- Working channel size
- Size of scope (outer shaft size F)
- Deflection ability in lower pole
- Location of working channel port on scope
- Deflection locking capability
- Image quality
- Weight
- Image display method
- Tip shape

**Question 15: if there was the possibility to improve three features on a single-use (disposable) flexible ureteroscope, which would it be? (Drag the top 3 features you prefer into the box to the right)**

- Addition of pressure sensor
- Small outer shaft size
- Larger deflection angle
- Improved optics and vision
- Improved irrigation outflow
- Addition of suction ability
- Addition of second working channel
- Wireless connectivity to image monitor
- Location of irrigation port
- Location of working port
- Addition of temperature sensor

**Question 16: Are there any other features you would want to improve on a single-use (disposable) flexible ureteroscopes beyond the list provided in the previous question?**

- No
- Yes[Describe]

**Question 17: How important is surgeon controlled image capture/recording buttons on single-use (disposable) flexible ureteroscope handle?**

- Essential
- Very important
- Somewhat important
- Not important
- Unnecessary

**Question 18: Please choose the figure (only one) where you think the optimal location of the working channel port entrance should be on a single-use flexible ureteroscope**

**
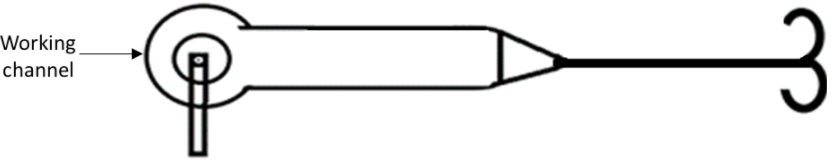
**

**
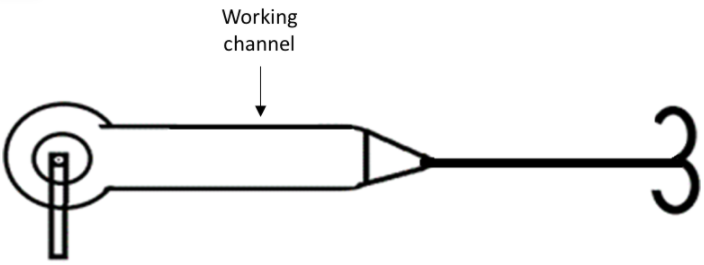
**

**Question 19: In your experience, what effect has single-use (disposable) flexible ureteroscope had on your operating room (OR) time?**

- Increase in OR time
- Decrease OR time
- No change in OR time

**Question 20: How important would be the incorporation of a temperature sensor into a single-use (disposable) flexible ureteroscope?**

- Essential
- Very important
- Somewhat important
- Not important
- Unnecessary

**Question 21: How important would be the incorporation of a pressure sensor into a single-use (disposable) flexible ureteroscope?**

- Essential
- Very important
- Somewhat important
- Not important
- Unnecessary

**Question 22: How important would be the ability to suction fluid, while maintaining adequate irrigation, with a single-use (disposable) flexible ureteroscope?**

- Essential
- Very important
- Somewhat important
- Not important
- Unnecessary

**Question 23: How important would be the ability to suction fragments during laser lithotripsy with a single-use (disposable) flexible ureteroscope?**

- Essential
- Very important
- Somewhat important
- Not important
- Unnecessary
